# Insight into the Molecular Imaging of Alzheimer's Disease

**DOI:** 10.1155/2016/7462014

**Published:** 2016-01-10

**Authors:** Abishek Arora, Neeta Bhagat

**Affiliations:** ^1^Amity Institute of Biotechnology, Amity University Uttar Pradesh, Noida 201303, India; ^2^Amity Institute of Biotechnology, Amity University Uttar Pradesh, Room No. 312, J3 Block, III Floor, Noida 201303, India

## Abstract

Alzheimer's disease is a complex neurodegenerative disease affecting millions of individuals worldwide. Earlier it was diagnosed only via clinical assessments and confirmed by postmortem brain histopathology. The development of validated biomarkers for Alzheimer's disease has given impetus to improve diagnostics and accelerate the development of new therapies. Functional imaging like positron emission tomography (PET), single photon emission computed tomography (SPECT), functional magnetic resonance imaging (fMRI), and proton magnetic resonance spectroscopy provides a means of detecting and characterising the regional changes in brain blood flow, metabolism, and receptor binding sites that are associated with Alzheimer's disease. Multimodal neuroimaging techniques have indicated changes in brain structure and metabolic activity, and an array of neurochemical variations that are associated with neurodegenerative diseases. Radiotracer-based PET and SPECT potentially provide sensitive, accurate methods for the early detection of disease. This paper presents a review of neuroimaging modalities like PET, SPECT, and selected imaging biomarkers/tracers used for the early diagnosis of AD. Neuroimaging with such biomarkers and tracers could achieve a much higher diagnostic accuracy for AD and related disorders in the future.

## 1. Introduction

A range of syndromes result in the destruction and loss of cells of the nervous system giving rise to various insidious but lethal neuropathies like Parkinsonism, Alzheimer's disease, Dementias, and Multiple Sclerosis. Such conditions are encompassed as neurodegenerative disorders [1]. The manifestation of such syndromes results in the degeneration of neurons, which ultimately culminates in the irreversible loss of neural function in the affected region of the brain [2, 3]. Neurodegenerative diseases induce characteristic impairments in the brain of the affected individual. These help in the characterisation and identification of specific neuropathies [4].

Advancements in the fields of clinical neuroscience have helped us in developing a deeper understanding of the induction as well as progression of neurodegenerative diseases [5]. The aggregation of misfolded proteins in various regions of the brain has been implicated in a majority of such neuropathies [6]. Despite various advancements in diagnostic techniques and the detailed study of molecules and subcellular process underlying such conditions, the neurological disorders are not well understood.

Conventionally, neurodegenerative disorders and allied syndromes were conclusively characterised at a late stage or via postmortem analysis [7]. The use of noninvasive techniques in medicine over the previous decade is popular owing to their ease of execution and increased patient well-being [8]. Molecular imaging has provided an alternative noninvasive tool for the diagnosis of neurological syndromes with high specificity in comparison to previous modalities [9]. The key advantage of molecular imaging modalities is due to its ability to elucidate sophisticated biological phenomenon at the cellular and molecular level, linking investigations to specific pathologies [10]. Also, molecular imaging makes it possible to provide information about changes before the pathological manifestation, which aids in the early diagnosis of neurological syndromes thereby allowing the timely implementation of appropriate therapeutic strategies [11].

There are various imaging modalities like magnetic resonance imaging (MRI) and Computerised Tomography (CT); however PET and SPECT are latest molecular imaging techniques that are extensively used in the diagnosis of neurological disorders [9, 12]. The molecular imaging procedure involves an imaging device and an imaging agent, or probe.

A variety of molecular probes are used to visualize the cellular activity and chemical processes involved in metabolism, oxygen distribution, or blood flow. Radiotracer atom or isotopes are also used for imaging the body. The imaging agent is introduced into the body, it accumulates at the target site, and its distribution is scanned, thus providing information about the changes taking place in the tissues and organs [13]. Commonly probes are used in the range of pico- to femtomoles per gram.

In the past decade, PET and SPECT were used to elucidate the neurochemical changes such as the role of neurotransmitters including dopamine, serotonin, and acetylcholine in neurodegenerative disorders. Recent technological advancements have enabled the use of these two techniques to probe a wide variety of intra- and extracellular proteins with impaired function and expression related to brain diseases. These advancements have enabled PET and SPECT to have applications from neurochemical imaging to molecular imaging, thereby elucidating various molecular pathophysiological processes of brain diseases.

The complete mechanism of neurodegenerative conditions has not yet been fully elucidated. Intense research in this field has identified as many as 500 novel molecular targets [14]. Novel molecular imaging agents like small molecules peptides, hormones, antibodies, aptamers, affibodies, transporter substrate nanoparticles, drugs, and oligonucleotides are used for the localization of such targets [15]. The use of novel compounds in PET and SPECT methods helps in diagnosing and understanding the pathophysiology involved in specific molecular changes that occur during the early stages of neurodegenerative disorders. In the present review, the applications of two imaging modalities, namely PET and SPECT, have been discussed in the molecular imaging of AD.

## 2. Pathophysiology and Biomarkers of Alzheimer Disease (AD)

Alzheimer's disease is an age-dependent neurodegenerative disorder that involves multiple molecular mechanisms. AD manifests as significant cognitive deficits, behavioural changes, sleep disorders, and loss of functional autonomy. The number of patients suffering from AD is growing rapidly worldwide. AD represents the foremost cause of Dementia and has become a major public health issue.

AD is a complex disorder which has many different pathophysiological features like impairment of cognitive domains, a characteristic pathological cortical and hippocampal atrophy, histological feature of senile plaques comprising of amyloid deposits and neurofibrillary tangles consisting of intraneuronal tau fibrillary tangles, and a resultant decrease in neurons. It is also accompanied by biochemical changes like abnormalities of cholesterol metabolism, inflammation, oxidative damage, and lysosomal dysfunction. Clinical diagnosis of AD remains difficult in initial stages. Current methods for diagnosing AD involve a detailed history and neuropsychological testing to establish the presence of Dementia. Other investigations must then be conducted to distinguish AD from other forms of Dementias such as Vascular Dementia (VaD), Frontotemporal Dementia (FTD), and Lewy Body Disease (LBD) [16, 17].

In AD, it is currently not possible to directly measure the number of remaining cortical neurons* in vivo* and, therefore, alternative approaches are required. Clinical assessments in AD using scales to measure cognitive impairment, disability, quality of life, or global disease severity are tarnished by symptomatic effects of therapy and are unable to differentiate this effect from disease-modification, at least in the short term. There is a dire need of AD biomarkers for both an early and accurate diagnosis and prediction of disease progression. Many candidate biomarkers for disease progression in AD have also been studied.

Several proteins like total tau (t-tau) and phosphorylated tau (p-tau) are “AD Signatures” which show marked increase in the cerebrospinal fluid (CSF). Other protein markers associated with AD are A*β*42, resistin, and thrombospondin-1 [18]. Mitochondrial dysfunction with degeneration of mitochondria in neurons [19], inflammatory mechanisms, oxidative stress [20], vascular homeostasis, altered lipid metabolism [21], and antioxidant defence system are some of the targets used for the diagnosis of AD. CSF proteome of AD patients shows altered levels of *α*-1-antitrypsin, *α*-1b glycoprotein, APOA-I, APOE, retinol binding protein, vitamin D-binding protein, prostaglandin H2 D isomerase, and transthyretin (TTR) [22–27]. CSF biomarkers of inflammation that showed increased levels in AD are TNF-*α* [28], monocyte chemotactic protein-1 [29], interferon *γ*-inducible protein 10, IL-8 [30], IL-6 [31], transforming growth factor-*β* (TGF*β*) [32], and vascular endothelial growth factor (VEGF) [31].

Several evidences have documented that cholesterol metabolism plays a role in AD [33]. Total serum cholesterol may be a marker of AD because high concentration of serum cholesterol is involved in tau phosphorylation and is caused due to the dysfunction of protein kinase C (PKC). The PKC function is involved in memory processes in animal models [34] and appears altered in red blood cells and lymphocytes of AD patients [35]. By inhibiting GSK3*β*, PKC reduces tau phosphorylation and neurofibrillary tangles formation [36] making it a potential target for drugs and the most promising marker in AD diagnosis. As many as 98 different proteins involved in oxidation reduction [37], glycolysis [38], transport [38], metabolic processes [16], protein folding [39], the response to unfolded proteins [40], and cell proliferation [40] have been reported to be associated with AD [41]. These proteins showed quantitative differences in AD and 56 of them are cytoplasmic, 28 mitochondrial, 20 nuclear, and 16 cytosolic proteins. Finally, three of them are synaptic proteins (synaptosomal-associated protein-25 (SNAP-25), synaptotagmin, and syntaxin-binding protein) which present altered expression or modification [42]. A decrease in the number of neurons, formation of amyloid plaques, and the generation of neurofibrillary tangles, which results in neuronal dysfunction, act as hallmarks of AD. Such recognition of CSF biological markers for AD gives an accurate “molecular” diagnosis and subsequent follow-up of the disease.

Biochemical biomarkers like arachidonic acid (AA) and docosahexaenoic acid (DHA), an omega-6 and omega-3 polyunsaturated fatty acid (PUFA), respectively, are very important constituents of phospholipids in cell membranes and contribute extensively to cell signalling in the brain. The CNS response to injury and to the onset (and progression) of neurodegeneration involves the release of free DHA and AA along with the synthesis of stereospecific docosanoid derivatives and prostanoids, respectively [43, 44]. Phospholipases, for example, PLA2, contribute to the conversion of AA into inflammatory molecules such as prostaglandin E2 (PGE2) by the cyclooxygenase (COX) 1 and 2 enzymes [45].

Protein biomarkers in the (CSF) such as a reduced amyloid or an elevated tau concentration have been used to diagnose early AD [38]. Lumbar puncture is an invasive procedure and may not be practically favourable for conducting large-scale studies on AD. Noninvasive neuroimaging methods such as positron emission tomography (PET) to measure amyloid in the brain or magnetic resonance imaging (MRI) to measure atrophy of medial temporal structures have also proved useful [46, 47].

However, PET is expensive and not readily available in many places, while brain atrophy, as measured by MRI, requires specialized facilities and is less specific to AD. The use of blood-based biomarkers is therefore an attractive alternative given the easy accessibility of blood [48]. Yet, there is a complex relationship between the different biomarkers. Putative biomarkers which are used in the diagnosis and prognosis of AD are positron emission tomography (PET) neuroimaging of *β*-amyloid (A*β*) protein deposition and magnetic resonance imaging (MRI) of hippocampal volume and other brain structures [49].

With the advent of high throughput techniques including transcriptome analysis and next generation sequencing methods, protein markers present in CSF and blood (i.e., plasma and serum) can be quantified accurately for diagnosis of AD. Extracellular plaques (A*β*42) and intracellular neurofibrillary tangles (tau) can be identified histopathologically and morphologically [50]. A*β*42, the main constituent of amyloid precursor protein (APP), is generated by sequential actions of *β*-secretase and *γ*-secretase on APP through an amyloidogenic pathway and there are several truncated A*β* isoforms in the brain [51].

Protein biomarkers involved in pathogenesis of AD are also identified by two-dimensional gel electrophoresis (2-DE) and matrix-assisted laser desorption/ionization combined with time of flight MS (MALDI-TOF-MS) and liquid chromatography combined with electrospray ionization (LC-ESIMS). In recent years, many new diagnostic tools like surface enhanced laser desorption ionization (SELDI-TOF-MS) which provides a high throughput protein expression profile analysis have evolved [52]. Isotope tagged relative and absolute quantitation (iTRAQ) [53], tandem mass tag (TMT) [54], isotope coded affinity tag (ICAT) [55], and isotope coded protein label (ICPL) [56] have been used for identification and quantification of proteins. Antibody array is another high throughput method to analyse multiple biomarkers [57].

The main tests for biomarkers classes used in the diagnosis and prognosis of AD are positron emission tomography (PET) neuroimaging of A*β* protein deposition and magnetic resonance imaging (MRI) of hippocampal volume and other brain structures [58]. These brain-imaging techniques are often used for studying the neuropathological processes and morphological and functional changes occurring in AD. Neuroimaging methods like PET and SPECT are helpful not only in the early diagnosis but also in differentiating AD from other neurodegenerative diseases.

## 3. Positron Emission Tomography (PET) in AD

Positron emission tomography (PET) is a nuclear medicine based molecular imaging technique that utilises a range of specially developed radiopharmaceuticals, which function as tracers. The technique is used to detect the rate of uptake of such tracers in specifically targeted cells throughout the body of the patient. The technique relies on the quantification of the radiotracer's decay, during which a positron is emitted, thereby generating a photon [7].

**Figure 1:**
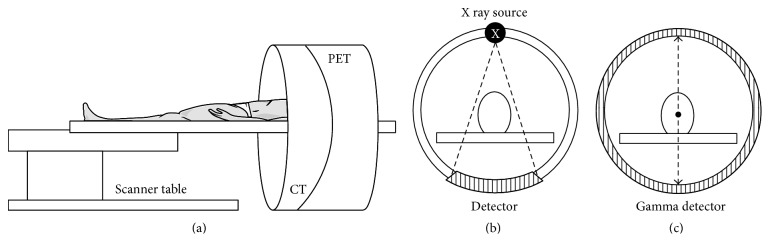
Schematic representation of (a) PET/CT scanner along with operational depiction of individual, (b) CT Module, and (c) PET Module of the scanning apparatus.

PET/CT thus functions as an* in vivo* imaging procedure that enables the study of systemic pathophysiological phenomenon, especially concerning neurodegenerative syndromes under its applications in the field of neurology [59]. The PET scanner detects pairs of energetic *γ*-rays that are indirectly emitted by the decay of the radiotracer that is administered to the patient. The radiotracer enters the brain via the blood brain barrier when administered intravenously. The radiotracer then accumulates in specific regions of the brain in accordance with the physiological condition that is being scrutinised [60]. The positrons emitted from the radiotracer transverse a few millimetres through the tissues in the vicinity of the neural vasculature transporting the radiotracer. This is accompanied with a rapid loss of kinetic energy of the traversing positrons. Further, the positrons travel slowly and interact with the electrons present in the neuronal cells to generate two 511 keV *γ*-rays travelling at an angle of approximately 180° to each other. This phenomenon is termed as annihilation [61]. The radiotracer utilised for the purpose of PET consists of a radiolabelled biologically active molecule that emits positrons at the time of undergoing decay [62]. The *γ* emissions of this radiotracer, followed by annihilation, are detected by the PET scanner, which generates three-dimensional views of the tracer localization within the patient's body (Figure 1).

The production of radiotracers utilised in PET requires the setting up of a specialised centre equipped with a significantly large cyclotron [63]. The production centre may be outsourced or on site depending on the half-life of the radiotracer being synthesised. Radiotracers that are used for PET based studies having extensive utilisation are labelled with ^18^F (*t*_1/2_ = 109.8 minutes), ^11^C (*t*_1/2_ = 20.3 minutes), or ^15^O (*t*_1/2_ = 2.04 minutes) [64]. The latter two must be produced at an on-site cyclotron owing to their short half-life. Generator based synthesis of radiotracers may also be done for labelling an active molecule with ^68^Ga (*t*_1/2_ = 67.83 minutes) and ^82^Rb (*t*_1/2_ = 1.27 minutes) [7].

Modern versions of the scanner are a hybrid between PET and CT technologies. The integration of functional imaging with structural imaging modalities plays a major role in attenuating the lacunae of anatomical acuity in the case of a simple PET based analysis. The PET/CT scanner is thus capable of generating anatomically augmented functional images of the brain [9]. By virtue of its high performance nature, PET has a wide variety of applications in the field of oncology, neurology, and cardiology [9]. The imaging modality has an extremely high sensitivity of nearly 10^−11^ to 10^−12^ mol/L and has an infinite depth of penetration [64]. The images obtained after the scan consist of PET and CT fusion images that show anatomical features along with a qualitative and quantitative distribution of the radiotracer in the brain. CT is helpful in the process of attenuation correction for accurate quantification and greater result reproducibility [65].

For the general process of image acquisition, the patient is asked to lie still in supine position on the scanner bed. The first step in image acquisition involves the initial action of performing a scout. Based on the protocol selected following the scout, a CT scan is performed which is followed by a PET scan of the defined region [66]. A brain PET/CT is performed for duration of 10 minutes without the administration of any IV contrast, which may be otherwise used in other investigations. An important pharmacological criterion that is applied in brain PET/CT based studies is that of binding potential (BP). The BP evaluates the density of neuronal receptors occupied by the radiotracer. Such an understanding aids in the characterisation of deviations in receptor localization which may further be pertinent for a particular neurological syndrome [67].

## 4. Single Photon Emission Computed Tomography (SPECT) in AD

Single Photon Emission Computed Tomography (SPECT) is a nuclear medicine modality that is related to PET in terms of utilising a radioactive tracer in order to elucidate the uptake of the radiotracer in the patient. However, unlike PET, the radiotracers used in SPECT directly emit *γ*-rays [68].

The radiotracers used in SPECT emit a single *γ*-ray at the time of each decay, which is directly detected by the *γ* camera of the SPECT scanner. The *γ* camera is rotated around the patient in order to record the emitted projections [7]. Depending on the configuration of the machine, it may consist of either a single headed or a double headed *γ* camera.

The *γ* camera is fitted with collimators in order to guide the emissions towards the *γ* cameras of the scanner [69]. The collimator that is used is composed of lead or tungsten, which rejects any emissions that are not propagated at right angles to the reference axis as specified at the time of the scan. This is important to detect the point of origin of the emission for accurate representation in the output image [70]. The collimator used in brain SPECT imaging is a low-energy high-resolution (LEHR) collimator.

**Figure 2:**
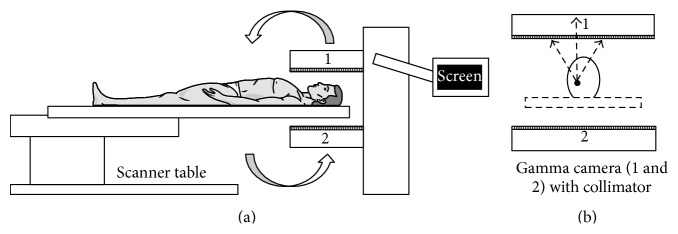
Schematic representation of (a) SPECT scanner along with depiction of (b) gamma camera placement and detection of emissions from a reference point at the time of a brain SPECT scan.

The radiotracers that are extensively used in the SPECT based investigation of neural function are labelled with ^123^I (*t*_1/2_ = 13.2 hours) and ^99m^Tc (*t*_1/2_ = 6.06 hours). The active molecule may also be labelled with ^67^Ga (*t*_1/2_ = 3.26 days) and ^111^In (*t*_1/2_ = 2.83 days) depending on the nature of analysis of the active molecule [71]. The principle of BP as discussed in PET/CT also applies to SPECT based molecular imaging. In lieu of the longer half-life of radiotracers utilised in SPECT, there is no requirement for an on-site cyclotron and a specialised radiochemistry facility. Such radiotracers are produced at a commercial scale. Owing to this, the lower expense of radiotracer generation for SPECT in comparison to PET/CT allows a wider and easily accessible utilisation of the scanning modality by patients. Brain SPECT image acquisition is performed by making the patient lie on the scanner bed in supine position. Appropriate positioning of the scanner bed and the *γ* cameras are done such that the collimators are in close proximity to the patient's head, while also allowing ease of movement for the *γ* cameras. The images obtained after the scan consist of SPECT images that show computed differential anatomical features along with a quantitative distribution of the radiotracer in the brain (Figure 2).

## 5. Radiotracers Used in PET and SPECT

A radiotracer used for neurological diagnostics must have optimal uptake, specific binding, and efficient clearance of the radiotracer [72]. The radiotracer being designed for diagnostics purposes must be of nontoxic and lipophilic nature [73]. It should have a low molecular weight so that it may easily transverse the blood brain barrier in order to enter the brain [74]. The radiotracer should be designed to reduce the incidences of nonspecific binding, should not get metabolised, and should be rapidly cleared from the blood [75]. The binding to its target must be specific and reversible in nature. The uptake of the radiotracer by the brain may further depend on a range of factors like permeability of the blood brain barrier, cerebral blood flow, plasma concentration of the radiotracer, and the noninteracting fractions of the radiotracer in the plasma and brain [74]. Furthermore, the selectivity of a particular neurological molecular imaging radiotracer is dependent on the concentration of vacant interaction site (Table 1) [73].

## 6. Radiotracers for Amyloid Imaging

**Figure 3:**
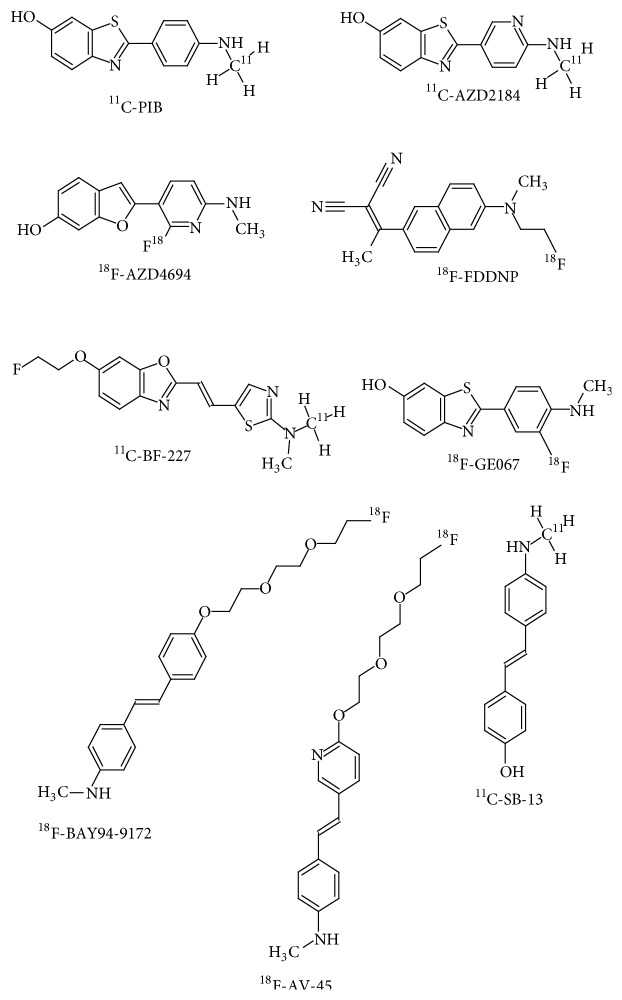
Structural representation of radiotracers for amyloid imaging.

The development of suitable radiotracers for the imaging of A*β* aggregates has been taking place over the previous decades [76]. Out of the various categories of the amyloid radiotracers undergoing trial at different stages, small molecule based radiotracers have been the most successful [77] (Figure 3). In the recent years, radiotracers having high specificity have been generated using A*β* antibodies and peptide fragments that have been labelled with a suitable radioactive moiety [78]. The further development of radiotracers based on stilbene, thioflavin [79], and acridine [80] aims to revolutionize A*β* molecular imaging strategies. A*β* specific neuroimaging radiotracers are of essential importance in the diagnosis of AD [81]. This may be attributed to the presence of moderate to severe aggregates of A*β* in the form of amyloid plaques in all patients of AD [82]. The amyloid plaques are known to develop several years prior to the actual manifestation of cognitive decline and amnesia that are characteristic for AD [83].

### 6.1. ^11^C-PIB

[^11^C]-2-[4-(Methylamino)phenyl]-1,3-benzothiazol-6-ol, also known as Pittsburgh Compound B, is the first PET based radiotracer that has been developed for specifically binding with fibrillar amyloid plaques [84] (Figure 3). Initial studies of the radiotracer in mice showed rapid uptake in the brain upon intravenous administration. The radiotracer also showed rapid clearance from healthy neuronal tissue while showing retention in cortex of AD brain [85]. The thioflavin-T derived lipophilic structural moiety of ^11^C-PIB is able to enter the brain via the blood brain barrier and displays sufficient specificity and high affinity to A*β* aggregates found in senile plaques [86]. In studies of diseased versus healthy controls, the localization of ^11^C-PIB after administration was found to be greater in the temporal, parietal, and frontal lobes. These findings were verified based on postmortem analysis of the same patient cohort. The areas where ^11^C-PIB accumulated in diseased patients corresponded with areas known to have higher A*β* concentrations [87]. The utilisation of ^11^C-PIB is helpful in the differential diagnosis of AD and other types of Dementias. Comparative studies have shown that patients with Frontotemporal Dementia show normal ^11^C-PIB uptake in a majority of clinical cases [81]. Patients that have been diagnosed with mild cognitive impairment show increased ^11^C-PIB uptake, which is comparable to the levels of ^11^C-PIB that have been observed in AD patients [88]. Likewise, a significant number of patients diagnosed with DLB also demonstrate an increase uptake of ^11^C-PIB [89]. ^11^C has a half-life of 20 minutes, limiting the utilisation of this radiotracer only in facilities that are equipped with an on-site cyclotron [90].

### 6.2. ^11^C-AZD2184

^11^C-AZD2184, [^11^C]-2-[6-(methylamino)pyridin-3-yl]-1,3-benzothiazol-6-ol, is another analogue of ^11^C-PIB, which has been synthesized by replacing the 2-phenyl moiety with a pyridine (Figure 3). The radiotracer binds to A*β* aggregates in amyloid plaques with considerable affinity and demonstrates decreased levels of nonspecific binding [91]. The structure of ^11^C-AZD2184 has a lower lipophilicity as compared to ^11^C-PIB [92]. Such a property further decreases the chances of nonspecific interactions of the radiotracer in the white matter of the brain in contrast to ^11^C-PIB [93].

### 6.3. ^18^F-FDDNP

2-[1-[6-[2-[^18^F]Fluoranylethyl(methyl)amino]naphthalen-2-yl]ethylidene]propanedinitrile (^18^F-FDDNP) is a PET imaging radiotracer developed for the visualization of senile plaques in AD [94] (Figure 3). The radiotracer is a small molecule that has affinity to amyloid plaques as well as neurofibrillary tangles in patients diagnosed with AD [95]. ^18^F-FDDNP is the first known radiotracer used in molecular imaging that has the ability to bind* in vivo* with amyloid plaques and NFTs, thereby making it possible to localize such aggregates in a noninvasive manner [96]. The first human brain PET images using ^18^F-FDDNP were obtained in an 82-year-old female who had been clinically diagnosed with AD [97]. The examination showed relative radiotracer clearance in different regions of the brain. However, the key findings were that the radiotracer had an affinity towards regions of significant A*β* as well as hyperphosphorylated tau aggregations, which were confirmed following an autopsy of the patient [98]. Another group indicated that ^18^F-FDDNP upon administration to AD patients in comparison to healthy controls has a higher residence time in the regions of the hippocampus, frontal lobe, parietal lobe, temporal lobe, and the occipital lobe [95]. Based on such findings it may be stated that the clearance time of ^18^F-FDDNP in various regions of the brain may be inversely correlated with the degree of cognitive impairment in patients that have been clinically diagnosed with AD [99].

### 6.4. ^18^F-AV-45 or ^18^F-Florbetapir

4-[(E)-2-[6-[2-[2-(2-[^18^F]Fluoranylethoxy)ethoxy]ethoxy]pyridin-3-yl]ethenyl]-N-methylaniline (^18^F-AV-45 or ^18^F-Florbetapir) is the first ^18^F labelled PET radiotracer that has been approved by the US Food and Drug Association for the clinical evaluation of patients suspected with AD and other allied syndromes of cognitive deterioration [100] (Figure 3).

^18^F-Florbetapir applications have been able to significantly replicate imaging findings that have been examined using ^11^C-PIB as an amyloid specific radiotracer [101, 102]. Additionally, an analysis of PET images that were obtained using ^18^F-Florbetapir in Phase 3 clinical trials has shown a significant correlation with A*β* distributions based on postmortem follow-ups of the trial patients [103].

### 6.5. ^18^F-BAY94-9172 or ^18^F-Florbetaben

4-[(E)-2-[4-[2-[2-(2-[^18^F]Fluoranylethoxy)ethoxy]ethoxy]phenyl]ethenyl]-N-methylaniline (^18^F-BAY94-9172 or ^18^F-Florbetaben) is an ^18^F labelled radiotracer used in PET based examinations of A*β* aggregates in AD and other forms of Dementia [104] (Figure 3). The cortical distribution of ^18^F-Florbetaben is considerably similar to that of ^11^C-PIB [105]. In a study that has attempted to differentiate AD from Dementia with Lewy Bodies (DLB) based on ^18^F-Florbetaben localization, the radiotracer demonstrated a lower overall retention in DLB patients in spite of a similar involvement of A*β* in the pathophysiology of DLB [83]. ^18^F-Florbetaben thus may play a substantial role in the differential diagnosis of Frontotemporal Dementia (FTD), Vascular Dementia (VaD), and Parkinson's disease (PD), in lieu of the absence of abnormal A*β* aggregations in such syndromes [89].

### 6.6. ^18^F-GE067 or ^18^F-Flutemetamol

2-[3-[^18^F]Fluoranyl-4-(methylamino)phenyl]-1,3-benzothiazol-6-ol (^18^F-Flutemetamol) is an amyloid radiotracer that is a structural analogue of ^11^C-PIB [91] (Figure 3). Initial studies in human subjects have shown that ^18^F-Flutemetamol has similar neuronal uptake as well as affinity to A*β* aggregates as seen in studies using ^11^C-PIB [106, 107]. ^18^F labelled A*β* specific radiotracers however showcase a higher nonspecific uptake in the white matter, which may also be visualized in the PET images of healthy controls [108]. The key disadvantage of such a class of amyloid imaging radiotracers is that they generate greater levels of nonspecific background noise in comparison to ^11^C-PIB [87].

### 6.7. ^18^F-AZD4694

2-[2-[^18^F]Fluoro-6-(methylamino)-3-pyridinyl]-1-benzofuran-5-ol (^18^F-AZD4694) has been developed so as to overcome the limitations of using ^11^C-AZD2184 as an amyloid specific radiotracer. On the basis of chemical characterisation, ^18^F-AZD4694 is an aromatic pyridinylbenzofuran that has undergone fluorosubstitution [74] (Figure 3). The uptake and distribution of ^18^F-AZD4694 are comparable with that of ^11^C-PIB [109]. By virtue of the shared structural similarity with ^11^C-PIB, ^18^F-AZD4694 thereby demonstrates similar pharmacodynamics as well as pharmacokinetics as ^11^C-PIB while at the same time overcoming the shortcomings of using ^11^C labelled radiotracers [110].

### 6.8. ^11^C-BF-227

[^11^C]2-(2-[2-Dimethylaminothiazol-5-yl]ethenyl)-6-(2-[fluoro]ethoxy)benzoxazole (^11^C-BF-227) is an optimized benzoazide derivative that is being analysed as a diagnostic radiotracer for *β*-amyloid aggregates [111] (Figure 3). ^11^C-BF-227 has demonstrated a good binding affinity for A*β* accompanied with efficient neurological uptake [112]. ^11^C-BF-227 localize in the frontal, temporal, lateral temporal, temporooccipital, anterior and posterior cingulate cortices, striatum, and the occipital areas of the brain where amyloid aggregates occur [111].

### 6.9. ^11^C-SB-13 or ^123^I-SB-13

[^11^C] 4-N-Methylamino-4-hydroxystilbene (^11^C-SB-13) is a stilbene-based derivative (Figure 3) that has selective affinity towards A*β* aggregates that are as previously mentioned observed as a constituent part of senile plaques in AD [113]. The radiotracer has similar* in vivo* properties as demonstrated by ^11^C-PIB, used for the diagnosis as well as prognosis of AD [114]. In human trials of the radiotracer initially conducted, ^11^C-SB-13 demonstrated significant levels of localization in known regions of A*β* accumulation as a part of AD pathogenesis [115]. This was possible due to efficient transport of the radiotracer across the blood brain barrier [116]. Rather, studies have indicated that the relative cortical uptake of ^11^C-SB-13 is greater than that of ^11^C-PIB [117]. ^11^C-SB-13 is more likely to interact with fibrillar A*β*; however further studies are required to establish the same [115]. Furthermore, the shape as well as dimensions of amyloid plaques determines the degree of penetration of the radiotracer [118]. A variant of the same radiotracer has been labelled with ^123^I. ^123^I-SB-13 has demonstrated effective SPECT applications in human trials; however its use warrants further analysis [119].

## 7. Radiotracers for Tau Imaging

The successful molecular imaging of A*β* using various developed radiotracers has given impetus to the development of tau specific radiotracers. The accumulation of hyperphosphorylated tau gives rise to neurofibrillary tangles (NFTs) [120]. However, such an aggregation occurs intracellularly among the nerve terminals [121]. This is in complete contrast to the extracellular formation of amyloid plaques [122]. In the NFTs, tau exists in the form of paired helical filaments (PHFs) [123]. The designing of tau specific radiotracers thus targets the PHF tau aggregates [124]. By virtue of the intracellular localization of PHF tau in affected neurons, it is difficult to generate tau specific radiotracers without certain affinity for A*β* [125]. Such an affinity towards tau may be incorporated in the radiotracer by introducing large hydrophilic moieties that may prevent interactions with A*β* [126]. A lot of initial work was focused on benzothiazole, pyrimidazole, and imadazothiazole derivatives as tau specific radiotracers [127]. Further onwards, efforts were made to characterise the use of oxindole, styryl benzimidazole, and thiohydantoin based tau radiotracers [128].

### 7.1. ^18^F-THK523

**Figure 4:**
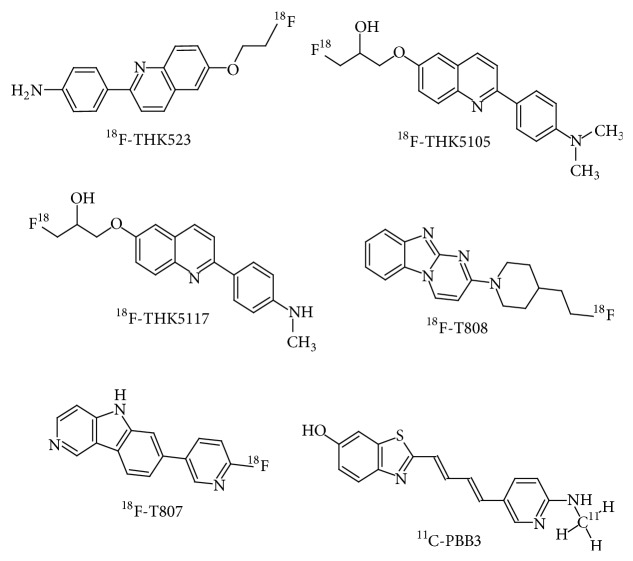
Structural representation of radiotracers for tau imaging.

2-(4-Aminophenyl)-6-(2-([^18^F]fluoroethoxy))quinoline (^18^F-THK523) is a quinolone-derived radiotracer (Figure 4) used in PET based examinations of PHF tau aggregates. Studies in the tau transgenic mouse model have shown that the radiotracer is able to enter the brain via the blood brain barrier and is able to bind with PHF tau aggregates [129]. Initial* in vivo* studies in humans have indicated greater levels of interaction of the radiotracer with PHF tau in comparison to A*β* [130].

*In vivo* examinations have demonstrated greater retention of the radiotracer in the orbitofrontal, parietal, hippocampal, lateral, and temporal regions in patients diagnosed with AD [131]. Furthermore, ^18^F-THK523 retention is not found to be associated with that of amyloid radiotracers of the likes of ^11^C-PIB [132]. Therefore, ^18^F-THK523 has selective affinity to tau aggregates. However, the localization of ^18^F-THK523 is lower in the grey matter in comparison to the white matter; this makes it difficult to examine such findings only based on visual inputs [131].

### 7.2. ^18^F-THK5105 and ^18^F-THK5117

6-[(3-^18^F-Fluoro-2-hydroxy)propoxy]-2-(4-dimethylaminophenyl)quinoline (^18^F-THK5105) and 6-[(3-^18^F-fluoro-2-hydroxy)propoxy]-2-(4-methylaminophenyl)quinoline (^18^F-THK5117) are 2-arylquinoline derivatives that have been labelled with ^18^F for PET based tau imaging [133] (Figure 4). These have been developed by further streamlining the binding and pharmacokinetics of ^18^F-THK523 [134].

Autoradiography based studies using ^18^F-THK5105 and ^18^F-THK5117 has shown their localization in the grey matter of the temporal lobe which correlates with the localization of PHF tau aggregates in the form of NFTs [133]. Human examinations using ^18^F-THK5105 via PET imaging have shown retention of the radiotracer in the lateral as well as mesial temporal lobes, which are otherwise known to have higher concentrations of tau aggregates in pathological cases [135]. Furthermore, the degree of retention of the radiotracer is significantly associated with the severity of Dementia and the degree of neuronal atrophy [135]. ^18^F-THK5117 is still a newer addition to the class of tau specific radiotracers and is being thoroughly analysed at various levels of function [136].

### 7.3. ^18^F-T807 and ^18^F-T808

The 7-(6-[^18^F]Fluoropyridin-3-yl)-5H-pyrido(4,3-b)indole (^18^F-T807) and 2-[4-(2-[^18^F]fluoranylethyl)piperidin-1-yl]pyrimido[1,2-a]benzimidazole (^18^F-T808) radiotracers were introduced after extensive autoradiography based studies of more than 900 compounds [137] (Figure 4). These radiotracers are mainly derivatives of benzimidazole that have a high affinity to PHF tau [138]. ^18^F-T807 PET based studies in AD patients have shown cortical localization of the radiotracer that is consistent with the known distribution of PHF tau in the brain [139]. Such findings are significantly coherent with postmortem features that correlate PHF tau distribution with the degree of disease severity [140]. Studies using ^18^F-T808 have shown faster pharmacokinetics as well as delayed defluorination of the radiotracer in comparison to ^18^F-T807 [141].

### 7.4. ^11^C-PBB3

The most recent member joining the ranks of other PHF tau specific radiotracers is 2-((1E,3E)-4-(6-(^11^C-methylamino)pyridin-3-yl)buta-1,3-dienyl)benzo[d]thiazol-6-ol (^11^C-PBB3), a phenyl/pyridinyl-butadienyl-benzothiazoles/benzothiazolium derivative (Figure 4). ^11^C-PBB3 demonstrated better visualization of tau aggregates in comparison to its predecessor ^11^C-PBB2 in mice models of AD, by virtue of which further work was carried out using ^11^C-PBB3 [142]. A human study using ^11^C-PBB3 demonstrated high affinity of the radiotracer to PHF tau aggregates [143]. However, significant localization of ^11^C-PBB3 was also noted in the venous sinuses of the subjects taking part in the same study [142]. This study also indicated that ^11^C-PBB3 has a low affinity to A*β* as the subjects involved in the study were also imaged with ^11^C-PIB. The localization patterns of both the radiotracers were consistently different such that individual correlations could be made with the known regions of aggregation of A*β* and PHF tau [143].

## 8. Radiotracers for Neuroinflammation

Neuroinflammation is a well-documented ageing associated phenomenon [144]. Neuroinflammation is a key player in the progression of neurodegenerative conditions and is known to occur during the early stages of onset of such syndromes [145]. The inflammation may be correlated with the activation of microglial cells in response to neuronal degradation in conditions including AD [146]. The molecular imaging of neuroinflammation may thus contribute to the characterisation of AD while also taking into consideration specific markers of AD pathophysiology [147].

### 8.1. ^11^C-PK11195 and ^123^I-Iodo-PK11195

**Figure 5:**
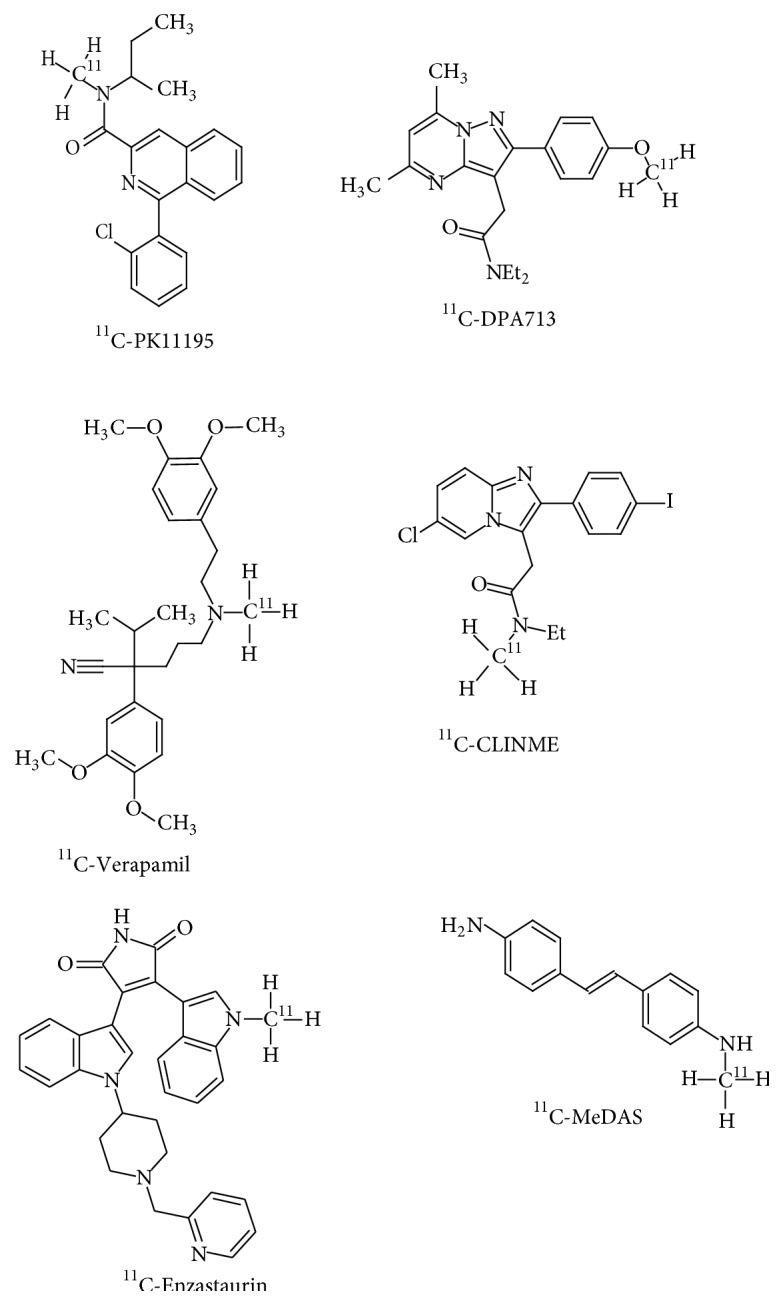
Structural representation of radiotracers for neuroinflammation and neuroprotection imaging.

The most successful radiotracer for PET based neuroinflammation studies is [^11^C]N-butan-2-yl-1-(2-chlorophenyl)-N-methylisoquinoline-3-carboxamide (^11^C-PK11195) (Figure 5). ^11^C-PK11195 specifically binds to the 18 kDa translocator protein (TSPO) also known as the peripheral benzodiazepine receptor (PBR) [148]. In normal physiological conditions, TSPO has only a basal expression in the microglial cells [149]. However, when the microglia undergo inflammatory activation, PBR-TSPO expression is upregulated, thereby functioning as a putative biomarker for neuroinflammation [150].

As per an initial study conducted to look into the clinical validation of ^11^C-PK11195 as a radiotracer for neuroinflammation, there was notably high localization of the radiotracer in the cingulate cortex, amygdala, fusiform gyrus, and the temporoparietal cortex of AD patients in contrast to similarly aged healthy controls [151]. ^123^I-Iodo-PK11195 is a modification of ^11^C-PK11195 for use in SPECT based imaging protocols. It functions as a high affinity ligand for PBR-TSPO [152]. As per a SPECT based study that was undertaken using this radiotracer, an increased retention of the radiotracer was observed in the temporal, parietal, occipital, and frontal lobes, wherein such findings were in tandem with AD induced neuroinflammation [153]. However, the use of PK11195 is limited for neuroinflammation imaging due to its increased incidences of nonspecific binding and lower neuronal bioavailability [154].

### 8.2. ^11^C-DPA713 and ^11^C-CLINME

[^11^C](N,N-Diethyl-2-[2-(4-methoxyphenyl)-5,7-dimethylpyrazolo[1,5-a]pyrimidin-3-yl]acetamide) (^11^C-DPA713) and [^11^C](2-[6-chloro-2-(4-iodophenyl)-imidazo[1,2-*α*]pyridine-3-yl]-N-ethyl-N-methyl-acetamide) (^11^C-CLINME) are new radiotracers that have been developed for the imaging of mild neuroinflammation [155] (Figure 5). Both the radiotracers have demonstrated a lower likelihood of nonspecific neuronal interactions and are sensitive to even low levels of TSPO expression due to their high affinity to the receptor [156]. ^11^C-DPA713 and ^11^C-CLINME were further optimized by labelling the ligands with ^18^F, thereby enhancing the half-life of the radiotracer. ^18^F-DPA714 is the successor of ^11^C-DPA713, showcasing better affinity and pharmacokinetics than PK11195 [157]. ^18^F-PBR111 is the fluorinated analogue of ^11^C-CLINME sharing properties that are inherent of the original radiotracer [158]. Such findings have found their basis in neuroinflammation studies that have been carried out in animal models of glioma and Multiple Sclerosis [159, 160]. ^123^I-CLINDE is another SPECT based radiotracer that has shown promising results in the preclinical examination of neuroinflammation [161]. The radiotracer retention appreciably correlates with the variations in TSPO that are observed at the onset and progression of neuroinflammation [162]. Another prominent feature of neuroinflammation and neurodegeneration is the phenomenon of astrocytosis [163]. Astrocytosis results in increased expression of imidazoline 2 binding sites (I_2_BS) [164]. Radiotracers that have been developed for the imaging of I_2_BS include ^11^C-DED and ^11^C-FTIMD. Current studies using ^11^C-DED in AD patients have shown increased radiotracer localization throughout the brain [165]. It has also been suggested that astrocytosis is a key feature of AD that functions as an intermediate between amyloidosis and neurodegeneration [166]. In case of ^11^C-FTIMD, animal model based studies have demonstrated that ^11^C-FTIMD has a high affinity to I_2_BS and has the ability to quantitate I_2_BS expression [167].

## 9. Other Molecular Radiotracers for Molecular Imaging

### 9.1. ^11^C-Enzastaurin

PKC as an enzyme is one of the most important initial elements involved in the induction of the previously mentioned *α*-secretases, ADAM-10 and 17, which are involved in neuroprotection. A potent and selective protein kinase C (PKC) inhibitor, Enzastaurin (LY317615), was recently labelled with ^11^C, thereby generating the radiotracer (3-(1-[^11^C]methyl-1H-indol-3-yl)-4-[1-[1-(2-pyridinylmethyl)-4-piperidinyl]-1H-indol-3-yl]-1H-pyrrole-2,5-dione), for PET imaging applications [168, 169] (Figure 5).

### 9.2. ^11^C-MeDAS

[^11^C]N-Methyl-4,4-diaminostilbene (^11^C-MeDAS) is a radiotracer, which can be used as a myelin-imaging marker for the early monitoring of myelin degeneration* in vivo*, and is a potentially useful development for the investigation of neurodegeneration [170] (Figure 5).

### 9.3. ^124^I-pQHNIG70

Impaired function of heat shock proteins HSP70, HSF1, and cathepsin proteins may facilitate the progression of neurodegeneration. The ^124^I-pQHNIG70 PET reporter system for imaging specific gene includes an inducible HSP70 promoter which can be used to image and monitor the activation of the heat shock factor 1 (HSF1)/HSP70 transcription factor on exposure to drug treatment 17-allylaminodemethoxygeldanamycin [171].

### 9.4. ^11^C-Verapamil

P-glycoprotein (P-gp) is a known BBB active efflux transporter involved in neuroprotection. Onset of PD and AD is characterised by its dysfunction [172]. The radiolabelled P-gp substrate 2-(3,4-dimethoxyphenyl)-5-[2-(3,4-dimethoxyphenyl)ethyl-[^11^C]methyl-amino]-2-propan-2-yl-pentanenitrile (^11^C-Verapamil) is used in PET studies of AD [173] (Figure 5).

### 9.5. ^11^C-AA and ^11^C-DHA

^11^C-Arachidonic acid (^11^C-AA) is incorporated in brain regions with neuroinflammation [174]. ^11^C-AA could thus be a novel marker of activated microglia to be used in studies of neurodegenerative disorders. Radiolabelled ^11^C-docosahexaenoic acid (^11^C-DHA) tracer is used to map the regional and global human brain DHA metabolism in relation to health and disease [175]. The quantitative imaging of DHA incorporation from plasma into the brain can be used as an* in vivo* biomarker of brain DHA metabolism and neurotransmission [176]. This may help to monitor DHA consumption* in vivo* in patients with disorders such as depression and AD, in which DHA supplementation may be helpful [177].

## 10. Conclusion

PET and SPECT with molecular probes are useful and reliable tools for clinical molecular neuroimaging. The methods have enabled* in vivo* assessment of molecular pathogenesis of CNS disorders. With these techniques, A*β* deposition, tau fibrillar mass, neurotransmitter turnover, and metabolism can be monitored accurately to better understand the pathological mechanisms underlying CNS diseases. In comparison to PET, SPECT is a more practical routine procedure for the detection of AD. But sensitivity, spatial resolution, and quantification of SPECT are limited. Improvements in a variety of molecular probes available for PET and SPECT will further help in identifying the biomarkers for biochemical processes underlying CNS diseases. In the forthcoming years, further advancements in imaging techniques promise to improve upon the early and accurate diagnosis, prognosis, and treatment of neurodegenerative diseases.

## Figures and Tables

**Table 1:** List of radiotracers designed for the PET as well as SPECT based analysis of AD pathophysiology.

Radiotracer	*t*_1/2_	Emission	Modality	Specificity	Condition
^11^C-PIB	20 minutes	Positron	PET	A*β*	AD
^11^C-AZD2184	20 minutes	Positron	PET	A*β*	AD
^18^F-FDDNP	110 minutes	Positron	PET	A*β* and Tau	AD
^18^F-AV-45	110 minutes	Positron	PET	A*β*	AD
^18^F-BAY94-9172	110 minutes	Positron	PET	A*β*	AD
^18^F-GE067	110 minutes	Positron	PET	A*β*	AD
^18^F-AZD4694	110 minutes	Positron	PET	A*β*	AD
^11^C-BF-227	20 minutes	Positron	PET	A*β*	AD
^11^C-SB-13	20 minutes	Positron	PET	A*β*	AD
^123^I-SB-13	13.2 hours	Gamma	SPECT	A*β*	AD
^18^F-THK523	110 minutes	Positron	PET	Tau	Tauopathies
^18^F-THK5105	110 minutes	Positron	PET	Tau	Tauopathies
^18^F-THK5107	110 minutes	Positron	PET	Tau	Tauopathies
^18^F-T807	110 minutes	Positron	PET	Tau	Tauopathies
^18^F-T808	110 minutes	Positron	PET	Tau	Tauopathies
^11^C-PBB3	20 minutes	Positron	PET	Tau	Tauopathies
^11^C-PK11195	20 minutes	Positron	PET	PBR-TSPO	Neuroinflammation
^123^I-PK11195	13.2 hours	Gamma	SPECT	PBR-TSPO	Neuroinflammation
^11^C-DPA713	20 minutes	Positron	PET	PBR-TSPO	Neuroinflammation
^11^C-CLINME	20 minutes	Positron	PET	PBR-TSPO	Neuroinflammation
^18^F-DPA714	110 minutes	Positron	PET	PBR-TSPO	Neuroinflammation
^18^F-PBR111	110 minutes	Positron	PET	PBR-TSPO	Neuroinflammation
^123^I-CLINDE	13.2 hours	Gamma	SPECT	PBR-TSPO	Neuroinflammation
^11^C-DED	20 minutes	Positron	PET	I_2_BS	Neuroinflammation
^11^C-FTIMD	20 minutes	Positron	PET	I_2_BS	Neuroinflammation
^11^C-AA	20 minutes	Positron	PET	AA analogue	Lipid metabolism
^11^C-DHA	20 minutes	Positron	PET	DHA analogue	Lipid metabolism
^11^C-Enzastaurin	20 minutes	Positron	PET	PKC	Neuroprotection
^11^C-MeDAS	20 minutes	Positron	PET	Myelin	Neuroprotection
^124^I-pQHNIG70	4.18 days	Positron	PET	HSF-1/HSP-70	Neuroprotection
^11^C-Verapamil	20 minutes	Positron	PET	P-gp	Neuroprotection
